# Genetic evidence supporting obesity as a risk factor for lung squamous cell carcinoma and the identification of *MFAP1* as a shared genetic target

**DOI:** 10.1007/s12672-026-04793-9

**Published:** 2026-03-12

**Authors:** Kai Xu, Tao Liu, Zhen Li, Manhua Wang, Zixuan Yang, Xinyu Chen, Yuqing Wang, Yizhou Chen, Yiyun Lin, Yu Wang, Gang Zhong, Xiaoqian Zhai

**Affiliations:** 1 Department of Thoracic Surgery, West China Hospital, Sichuan University, Chengdu, Sichuan China; 2 Lung Cancer Center/Lung Cancer Institute, West China Hospital, Sichuan University, No. 37 Guoxue Alley, Chengdu, 610041 Sichuan PR China; 3 Department of Thoracic Surgery, Japan Friendship Hospital, Beijing, China; 4 West China School of Medicine, Sichuan University, Chengdu, Sichuan China; 5 West China School of Basic Medical Sciences & Forensic Medicine, Sichuan University, Chengdu, Sichuan China; 6 College of Life Sciences, Sichuan University, Chengdu, Sichuan China; 7 Montefiore Medical Center, Albeit Einstein College of Medicine, Bronx, NY USA; 8 Neuroscience Institute, Carnegie Mellon University, 4400 Fifth Ave, Pittsburgh, PA 15213 USA; 9 Graduate School of Biomedical Sciences, University of Texas MD Anderson Cancer Center, Houston, TX USA; 10 Department of Mathematics, University of Georgia, 200 D. W. Brooks Drive, Athens, GA 30602 USA; 11 Department of Orthopedic Surgery, West China Hospital, Sichuan University, Chengdu, Sichuan China; 12 Trauma Center, West China Hospital, Sichuan University, Chengdu, Sichuan China; 13 Department of Medical Oncology, Cancer Centre, West China Hospital, Sichuan University, Chengdu, Sichuan China; 14 Department of Mathematics, Louisiana State University, Baton Rouge, LA 70802 USA

**Keywords:** Lung squamous cell carcinoma, Obesity, Mendelian randomization, Genetic correlation, MFAP1, Multi-omics

## Abstract

**Background:**

Observational studies have suggested that obesity may protect against lung cancer, particularly lung squamous cell carcinoma (LUSC). However, these findings are likely influenced by confounding factors such as smoking. We aimed to clarify the genetic relationship between body mass index (BMI) and LUSC and to identify potential shared therapeutic targets.

**Methods:**

We combined several genome-wide approaches to investigate shared genetic architecture between BMI and LUSC, including genetic correlation analyses and bidirectional Mendelian randomization (MR). We then examined tissue- and cell-level heritability enrichment and used gene-level integrative analyses to prioritize functional candidates. Finally, phenome-wide MR was applied to explore potential clinical consequences of targeting key genes.

**Results:**

BMI and LUSC showed a significant positive genetic correlation. MR analyses provided genetic evidence consistent with acausal effect of higher BMI on increased LUSC risk. Genetic signals were enriched in selected tissues and cell types, including epithelial and neuronal populations. Genetic signals were enriched in selected tissues and cell types, including epithelial and neuronal populations. Across SMR and colocalization analyses, MFAP1 emerged as a shared functional gene linking BMI and LUSC. Phenome-wide MR identified limited but notable associations between higher MFAP1 levels and connective tissue–related and other clinical phenotypes, suggesting that potential on-target effects would need to be considered carefully.

**Conclusions:**

This study supports obesity as a genetic risk factor for LUSC and highlights *MFAP1* as a potential shared target at the interface of adiposity and squamous lung carcinogenesis. These findings provide a genetic framework for understanding obesity-related LUSC risk and offer hypotheses for future mechanistic and translational research.

**Supplementary Information:**

The online version contains supplementary material available at 10.1007/s12672-026-04793-9.

## Introduction

Globally, the incidence of lung cancer is rising, making it the leading cause of cancer-related deaths. Non-small cell lung cancer(NSCLC) accounts for the majority of all lung cancers (about 80%), of which 20%-30% are squamous subtype [1]. The therapeutic options for LUSC remain limited, posing significant challenges for managing advanced-stage patients [2].

Obesity is a worldwide health problem and observational studies have established associations between obesity and at least 11 cancer sites [3]. However, high BMI appears to act as a protective factor for lung cancer in observational studies, a phenomenon termed “obesity paradox”, which also applies to LUSC [4, 5]. It’s hard for observational studies to fully account for confounding factors such as smoking, because conventionally measured environmental exposures are often related to multiple behavioral, social and physiological factors that confound associations with outcomes [6]. Two-sample MR is a reliable approach for exploring potential causal associations while reducing the influence of confounding factors [7]. In contrast to the obesity paradox, MR analysis have reported that increased BMI raised the risk of LUSC [8–10]. However, the exact genetic connections between obesity and LUSC still need to be discovered and confirmed, as this may facilitate to the development of strategies targeting both LUSC and obesity and identification of risk loci [11, 12]. Recent multi-omics and “next-generation cancer phenomics” frameworks integrating genomic, transcriptomic, and clinical features have been proposed to dissect the molecular heterogeneity of lung cancer and to identify novel biomarkers and therapeutic targets [13–15].

In this study, we obtained GWAS summary data for LUSC patients and BMI and applied bidirectional MR to elucidate their causal relationship. We also explored their genetic correlation and shared heredity architecture. Finally, we identified a potential shared hub gene and explored its druggable potential and possible side effects of treatment, which may provide insights for the prevention and treatment of LUSC.

## Materials and methods

### Datasets

#### GWAS summary statistics

The GWAS summary data were obtained from the MRC-IEU online database (https://gwas.mrcieu.ac.uk/). The GWAS summary data for BMI was derived from a large meta-analysis of European-ancestry cohorts [16]. The GWAS summary results of LUSC were obtained from derived from the Transdisciplinary Research in Cancer of the Lung (TRICL) study, which including 54,763 controls and 7704 LUSC cases of European origin.

#### Bulk-tissue RNA sequencing gene expression data

We obtained bulk-tissue RNA sequencing data for LDSC-Specifically Expressed Gene (LDSC- SEG). Summary-data-based mendelian randomization (SMR) analyses were based on summary data from the Genotype-Tissue Expression (GTEx) project, a public data resource for gene expression in 53 primary tissues from unaffected individuals. Whole-blood cis-eQTL summary statistics were eQTLGen phase II (https://eqtlgen.org/), a meta-analysis of 14,115 individuals.

#### Single-cell RNA sequencing gene expression data

We obtained four single-cell RNA sequencing (scRNA-seq) datasets from brain (GSE162631), colon (GSE166555), small intestine terminal ileum (GSE242086), heart (SCP498, Single Cell Portal). The “EWCE” and “SingleR” R package were used to process the scRNA-seq data.

### Statistics

#### Flowchart of this MR-based study

**Fig. 1:**
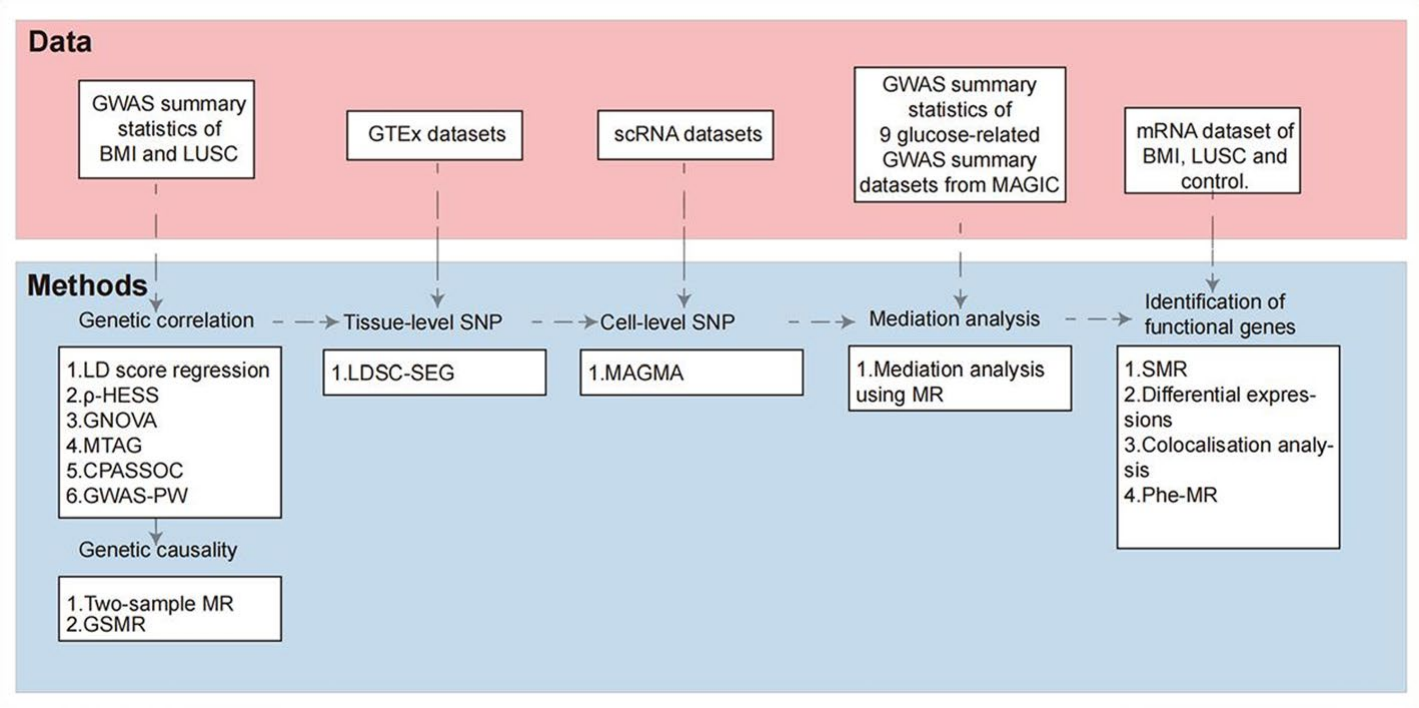
Flowchart Overview of this MR-based study. *LD* linkage disequilibrium, *ρ-HESS* Heritability Estimation from Summary Statistics, *GNOVA* Genetic covariance analyser, *MTAG* Multi-Trait Analysis of GWAS, *CPASSOC* Cross Phenotype Association, *GWAS* Genome-wide Association Study, *MR* Mendelian Randomization, *GSMR* Generalized summary-data-based Mendelian randomization, *LDSC-SEG* linkage disequilibrium score regression applied to specifically expressed genes, *MAGMA* Multi-marker Analysis of GenoMic Annotation, *SMR* Summary-databased Mendelian randomization, *Phe-MR* phenotypes, *MR BMI* body mass index, *LUSC* squamous cell lung cancer; *scRNA-seq* single-cell RNA sequencing

Using GWAS summary statistics (Fig. 1), we first investigated the genome-wide genetic correlation between BMI and LUSC using LDSC, ρ-HESS, and GNOVA. We then used multi-trait analysis of GWAS (MTAG), cross-phenotype association test (CPASSOC) and the pairwise GWAS Method (GWAS-PW) to investigate local genetic correlations between BMI and LUSC. Based on evidence of genetic correlation, we next estimated the genetic causality using two-sample MR and generalised summary-data-based MR (GSMR). To explore which organs and cell types would may coordinate the relationship between BMI and LUSC, we further examined SNP heritability enrichment at the tissue and cellular levels. At the tissue-level, we used LDSC-SEG (linkage disequilibrium score regression applied to specifically expressed genes) to identify specific tissues in which genes with increased expression were enriched for SNPs, using the GTEx datasets. At the cell-level, the scRNA-seq datasets were processed using the MAGMA. Finally, we applied SMR and colocalization analyses to identify shared functional genes and used phenome-wide MR (Phe-MR) to evaluate the possible side effects of targeting these genes.

#### Heritability and genetic correlation

LDSC is a commonly used method for estimating SNP heritability and genetic correlations between complex diseases and traits [17]. We calculated LD scores based on data from 1000 Genomes Project and used these scores as criteria to filter out SNPs with MAF ≤ 0.01 or INFO score ≤ 0.9 [18]. We then used stratified LDSC (S-LDSC) to estimate SNP heritability for BMI and LUSC and bivariate LDSC to estimate the genetic correlation between BMI and LUSC [19, 20]. As a sensitivity analysis, we repeated LDSC with constrained intercepts. Specifically, we set the single-trait heritability intercepts for BMI and LUSC to 1 and the cross-trait intercept to 0, as there may be mild sample overlap between the two traits.

GNOVA is an approach that allows estimation of SNP-based heritabilities and genetic correlations between tow phenotypes, and provides higher precision and improved statistical inference compared with LDSC in some settings [21]. We used GWAS data for BMI and LUSC to validate the genetic correlation between the two traits, based on European population reference data from the 1000 Genomes Project and using default parameters.

To account for potential sample overlap between the BMI and LUSC GWAS datasets, we additionally compared LDSC estimates obtained with unconstrained and constrained intercepts, and interpreted the cross-trait genetic correlation in conjunction with these sensitivity settings.

#### Cross-trait meta-analysis

MTAG and CPASSOC are two cross-trait characterization methods used to detect shared risk SNPs for BMI and LUSC [22–24]. MTAG is a computationally efficient method for joint multi-phenotype analyses using GWAS summary statistics [22]. We used MTAG to calculate each SNP’s effect size for BMI and LUSC based on the following criteria MAF ≥ 0.01, sample size N ≥ (2/3) × 90th percentile, and genome-wide significance defined as P-value < 5 × 10^− 8^. To assess the potential inflation of false positives, we calculated an upper bound on the false discovery rate (‘maxFDR’). CPASSOC is a general method for detecting cross-phenotypic associations and was used as a sensitivity analysis [24]. We used the Shet statistic of CPASSOC to calculate the associations between SNPs and BMI/LUSC, and the SNPs with P-value < 5 × 10^− 8^ were considered significant.

#### Identification of local genetic correlations

ρ-HESS can estimate and visualize local SNP heritability and genetic correlations from GWAS summary association data [12]. Using ρ-HESS, we analyzed whether BMI and LUSC are genetically associated within specific genomic regions. Following previous work, we divided the genome into 1699 approximately independent regions, each of about 1.5 MB [25]. For each region, we calculate the local SNP heritability for BMI and LUSC and the local genetic correlation between the two traits, using the 1000 Genomes Project as the LD reference.

To validate shared genomic regions between BMI and LUSC, we used the pairwise GWAS (GWAS-PW) method [26]. GWAS-PW estimates a posterior probability for regions shared by the two traits (PPA3) or regions associated with both traits but driven by different causal variants (PPA4). We considered regions with PPA4 > 0.8 as having strong evidence for shared association.

#### Mendelian randomization

We applied the R packages “TwoSampleMR” and “GSMR” and the GCTA software to further explore whether there is causal effect between BMI and LUSC (*P*<0.05) [27]. The MR analysis included five complementary methods: MR-Egger, inverse variance weighting (IVW), weighted median, weighted mode, and GSMR. IVW and GSMR were considered the primary methods in our study [28]. We applied the MR-Egger intercept test, Cochran’s Q statistic test and leave-one-out tests as sensitive analysis to evaluate potential horizontal pleiotropy and the influence of individual SNPs [29].

Briefly, instrumental variants were selected according to the three assumptions [7]. We selected SNPs associated with the exposure trait (BMI or LUSC) at genome-wide significance (*P* ≤ 5 × 10^− 8^) as instrumental variables (IVs). To ensure independence of instruments, we then performed linkage disequilibrium (LD) clumping using the “clump_data” procedure in TwoSampleMR, with an r² cutoff of < 0.001 and a clumping window of 10,000 kb. LD was estimated with reference to the 1000 Genomes Project European panel, which is consistent with the ancestry of the GWAS summary statistics used in this study. When harmonizing exposure and outcome datasets, palindromic SNPs with ambiguous strand alignment were removed.

For each selected SNP, e calculated the F statistic using the conventional formula F = β²/SE². SNPs with F < 10 were considered weak instruments and excluded from further analyses [30]. For GSMR, we additionally applied the HEIDI (Heterogeneity in Dependent Instrument)-outlier procedure to remove SNPs with evidence of pleiotropy.

#### LDSC-SEG analysis

LDSC-SEG was applied to explore tissue-specific SNP heritability enrichment for both BMI and LDSC [31]. We used the 1000 Genomes Phase 3 European-ancestry data as the reference panel to evaluate LD scores. SNPs in HapMap 3 with MAF>0.05 were selected for analysis. Genes from the GTEx project were ranked according to a t statistic derived from the baseline model and all gene sets. The ranked genes reflected tissue-specific expression patterns across 53 tissues. From this ranked list, the top 10% specifically expressed genes with the highest t-statistic were selected to evaluate the SNP heritability enrichment in different tissues.

We then calculated the coefficient P-values based on the corresponding Z score. A coefficient P-value < 0.05 was used as the threshold for identifying the tissues with significant enrichment of SNP heritability for BMI and LUSC.

#### Cell type enrichment analyses using scRNA-seq datasets

MAGMA cell typing was conducted to estimate the cell type–specific expression and the genetic association between BMI/LUSC and gene expression. We estimated the cell-type enrichment across the four tissues (heart, ileum, brain and colon) using MAGMA and considered P-value< 0.05 as evidence of significant enrichment. We also conducted the Expression Weighted Cell Type Enrichment (EWCE) and the MAGMA_Celltyping R package to calculate the cell type specificity matrices for scRNA-seq datasets used in MAGMA analyses [32].

#### Summary-data-based Mendelian randomization

SMR is a technique used to determine associations between genetically determining traits and outcomes of interest [23]. We conducted SMR analysis to identify candidate risk genes with potential causal effects on BMI and LUSC by analyzing GWAS and eQTL summary statistics [33]. Genomic expression data sourced from GTEx V8 and cis-eQTL summary data from eQTLGen were utilized for the eQTL expression analysis of whole blood [34].

By default, SNPs were excluded if they exhibited strong LD (r² > 0.9) with the top eQTLs and had a minor allele frequency (MAF) < 0.01. The top associated variants were identified based on expression probes with eQTL *P* ≤ 5 × 10^− 8^. All SNPs were extracted by genome-wide complex trait analysis (GCTA) software to ensure their independence [27]. We used the HEIDI test to distinguish linkage from pleiotropy; HEIDI P-values > 0.05 were considered to indicate homogeneity and support a single shared causal variant.

To control for multiple testing in the SMR analyses, we applied a Bonferroni correction based on the number of tested expression probes. Specifically, a probe was considered statistically significant if its SMR P-value was below 0.05 divided by the number of probes tested. Throughout the manuscript, SMR results described as “significant” correspond to this Bonferroni-corrected threshold, unless otherwise specified.

#### Colocalization analysis

Colocalization analysis was used to address the limitations related to LD and pleiotropy in MR by formally evaluating whether BMI, LUSC, and gene expression signals share a common causal variant. Using the R package COLOC (v5.2.2) [35], we examined the colocalization between the eQTL signals for candidate genes (*MFAP1*, *CYP21A2*, *EPHB1*, *PRSS16*, *ZSCAN16*, *ZKSCAN8* and *PDIA3*) and GWAS signals for BMI and LUSC, respectively.

The COLOC evaluates five mutually exclusive five hypotheses: PPH0, no association with either trait; PPH1, with association solely with the expression of the gene; PPH2, association only with the disease/trait; PPH3, association with both traits but driven by distinct causal variants; PPH4, association with both traits driven by a shared causal variant [36]. A PPH4 of > 0.8 shows that there is strong colocalization, and medium colocalization indication is defined as 0.5 < PPH4 < 0.8 [37].

#### Phenome-wide MR

A phenome- MR analyze was performed to explore potential side effects associated with targeting *MFAP1* at the mRNA and protein levels [38]. We use publicly available pQTL data from Iceland 4907 plasma proteomics study and cis-eQTL data from eQTLGen phase II to instrument *MFAP1* protein and transcript levels, respectively (https://eqtlgen.org/). As outcome traits, and following the strategy described by Su’s study, we selected GWAS summary statistics for 783 disease-related phenotypes with at least 500 cases from the SAIGE GWAS resource (https://www.leelabsg.org/resources) [38].

For each outcome, two-sample MR analyses were conducted using MFAP1-related instruments. To address the issue of multiple testing across the large number of phenotypes, we controlled the false discovery rate (FDR) using the Benjamini–Hochberg procedure. Associations were considered statistically significant if they met an FDR-adjusted P-value < 0.05. In the main text, we primarily report phenome-wide MR findings that surpassed this FDR threshold, and we describe other associations as exploratory.

### Ethics approval statement

The need for research ethics approval was waived because the data were available with open access.

## Results

### Positive genetic correlations between BMI and LUSC

**Fig. 2:**
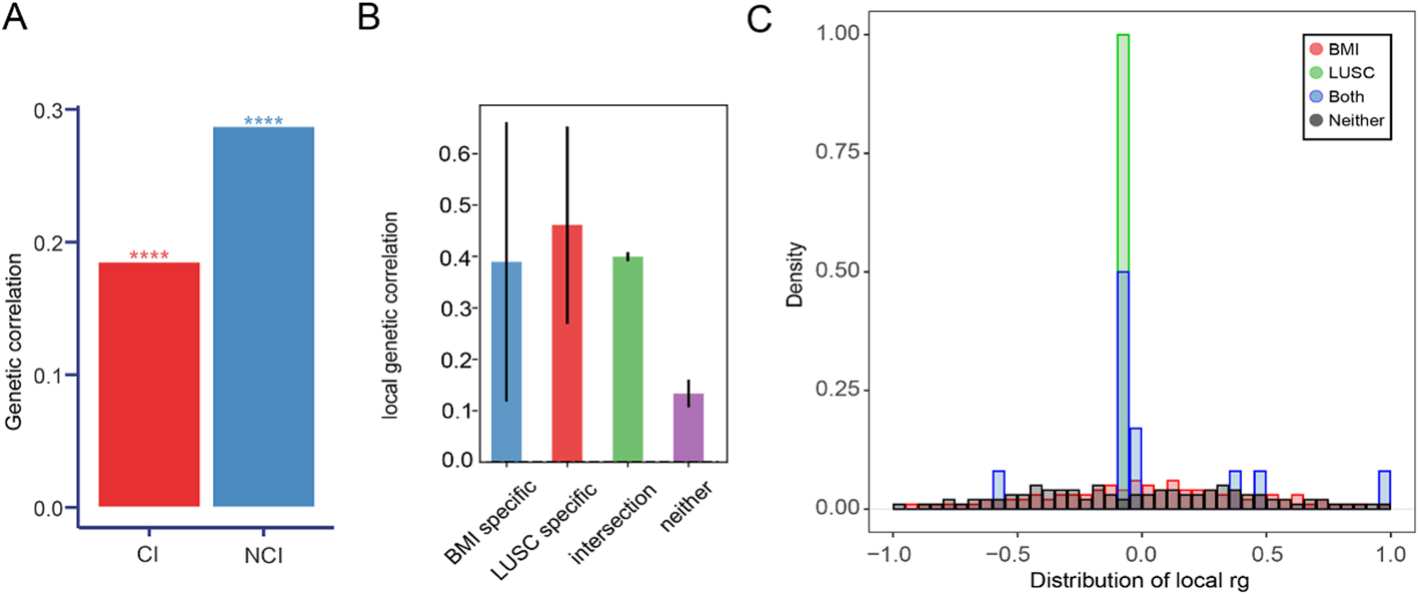
Genome-wide and local genetic correlations (rg) between BMI and LUSC. **A** Local genetic correlation estimates (rg) between BMI and LUSC across 1,699 approximately LD-independent genomic regions, calculated using bivariate LDSC. Each point represents a genomic region; the x-axis denotes the genomic position (ordered along the autosomes), and the y-axis denotes the estimated local rg. Regions with positive and negative local rg are shown above and below zero, respectively. **B** Mean local rg estimates for BMI and LUSC stratified by region category: regions harboring BMI-specific risk variants, regions harboring LUSC-specific risk variants, regions harboring shared risk variants (“intersection”), and all other regions (“neither”). The x-axis shows the four region categories, and the y-axis shows the average local rg. Error bars represent 95% confidence intervals, calculated using the jackknife method. **C** Density distributions of local rg estimates for BMI and LUSC across the same region categories. The x-axis shows local rg values and the y-axis shows the density. Curves for BMI-specific, LUSC-specific, intersection, and neither regions are displayed in different colors to highlight differences in the distribution of local genetic correlations. *BMI* body mass index; *LUSC* lung squamous cell carcinoma, *LDSC* linkage disequilibrium score regression, *rg* genetic correlation

First, we used bivariate LDSC to estimate the genome-wide genetic correlation (without constrained intercept) between BMI and LUSC. As shown in Fig. 2 and Table S1, we observed a significant positive genetic correlation (rg = 0.19, *p* = 5.69 × 10–25). The liability-scale SNP heritability estimates were 20.9% for BMI and 5.6% for LUSC, respectively, as summarized in Table 1. When we repeated LDSC with the intercept constrained under the assumption of no sample overlap, the genetic correlation remained significant (rg = 0.29, *p* = 2.58 × 10^− 10^, Fig. 2A and Table S1), supporting the robustness of the finding. Consistent with LDSC, GNOVA also showed a positive and significant genetic correlation between BMI and LUSC (rg = 0.219, *P* = 1.27 × 10⁻¹⁵; Table 1), further confirming a shared genetic basis between the two traits.

**Table 1:** Heritability and genetic correlation between body mass index and squamous cell lung cancer

Heritability h2	Method	BMI	LUSC
	LDSC	0.209	0.056
Heritability h2	GNOVA	0.238	0.161
Heritability h2	ρ-HESS	0.259	0.219
			P-value
Genetic correlation r_g_	LDSC	0.287	2.58E-10
Genetic correlation r_g_	GNOVA	0.219	1.27E-15
Genetic correlation r_g_	ρ-HESS	0.137	1.55E-24

*BMI *body mass index,*LUSC *squamous cell lung cancer

### Local genetic correlation further revealed the genetic relationship between BMI and LUSC on chromosomes 3, 6 and 15

Given the strong genome-wide genetic relationship between BMI and LUSC, we next explored local genetic correlations. Using MTAG and CPASSOC, we identified 350 genome-wide shared SNPs (*p* < 5 × 10^− 8^; Table S2). We further validated these shared loci using ρ-HESS and GWAS-PW, and found that 97 SNPs remained significant with PPA4 > 0.8 (Table S3). The maxFDR values of MTAG analysis for BMI and LUSC were 6.41 × 10 − 6 and 0.146, respectively indicating acceptable control of false discovery.

To further characterize the shared genetic regions, we employed ρ-HESS and identified three genomic regions with significant local genetic correlations: chromosome 3 (49316972–51832015), chromosome 6 (25684587–26791233), and chromosome 15 (67094767–69017999) (Table S4). As illustrated in Fig. 2A and Figure S1, these regions show markedly elevated local rg compared with the genome-wide background. The local single-trait SNP heritability estimates for BMI and LUSC were 25.9% and 21.9%, respectively, in regions harboring BMI-specific or LUSC-specific loci (Fig. 2B), and the density distribution of local rg further highlights the enrichment of shared genetic effects in “intersection” regions (Fig. 2C). Overall, the local heritability patterns reinforce the presence of shared genetic architecture between BMI and LUSC.

### MR showed causal relationship between BMI and LUSC

**Fig. 3:**
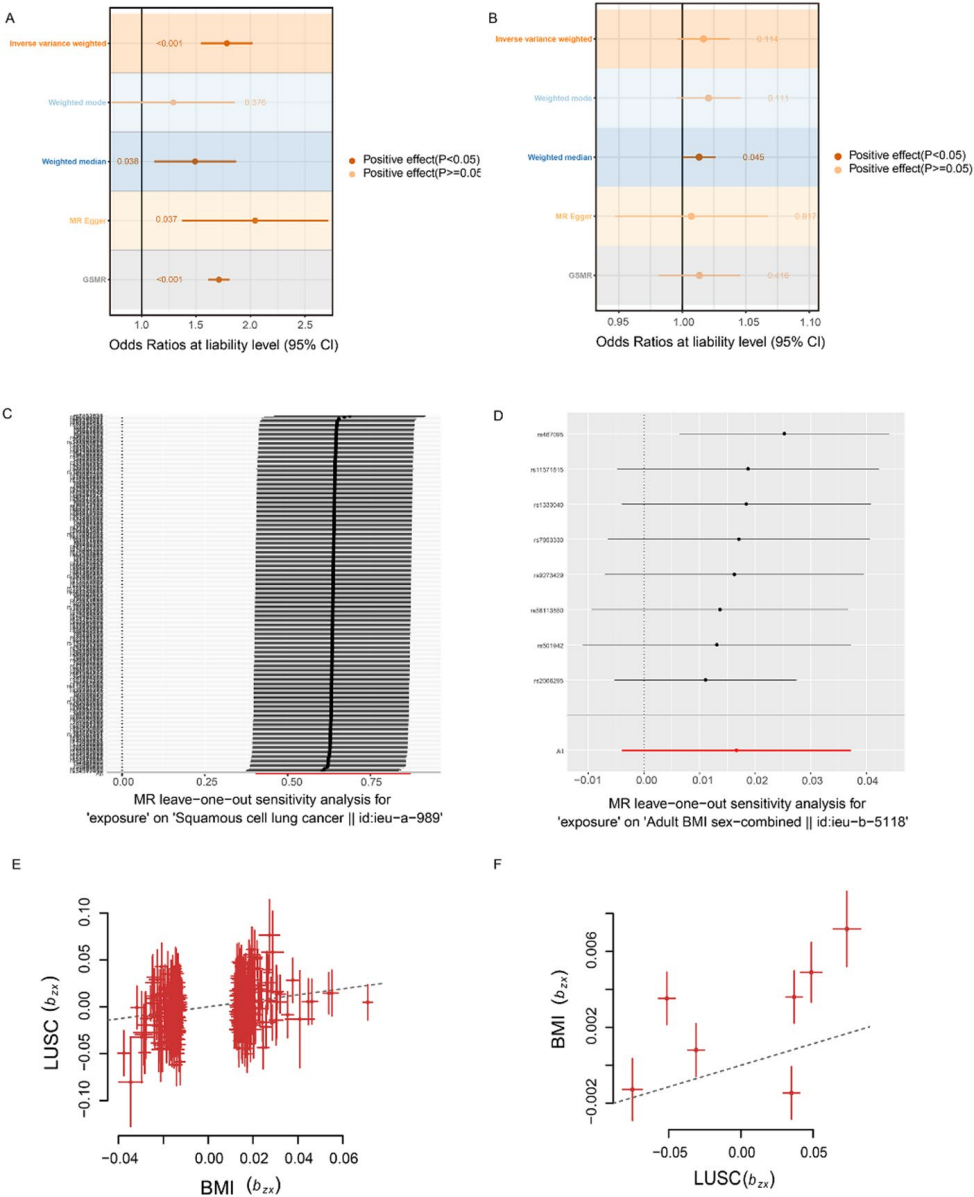
The forest-plot of bi-directional MR analyses between BMI and LUSC and leave-one-out test. **A** Causality of BMI on LUSC. **B** Causality of LUSC on BMI. **C** Leave-one-out analysis for BMI on LUSC; **D** Leave-one-out analysis for LUSC on BMI. **E** GSMR of BMI on LUSC; **F** GSMR of LUSC on BMI. *BMI* body mass index, *LUSC* Lung squamous cell carcinoma, *GSMR* generalized summary-data-based mendelian randomization

Given the evidence of genetic correlation, we further explored whether there is a causal relationship consistent with a causal influence of using bi-directional MR. IVs were selected according to standard MR assumptions and clumped as described in the Methods (Table S5, S6). As shown in Fig. 3A and Table S7, the IVW and GSMR analyses indicated that higher genetically predicted BMI was associated with an increased risk of LUSC (IVW β = 0.579, SE = 0.121, *P* = 1.83 × 10⁻⁶; GSMR β = 0.537, SE = 0.050, *p* = 5.45 × 10⁻²⁷.

The leave-one-out analysis for BMI on LUSC (Fig. 3C) demonstrated that single SNP do not make influence to the effect significantly, supporting the stability of the MR estimate. In contrast, the reverse MR analysis using LUSC as the exposure and BMI as the outcome did not provide evidence for a genetic effect consistent with causality (Fig. 3B and D; Table S7). GSMR results for both directions are also summarized in Fig. 3E–F, further supporting a directional effect from BMI to LUSC rather than the reverse.

### Tissue-level SNP heritability enrichment showed co-enrichment of SNPs from BMI and LUSC in the brain

**Fig. 4:**
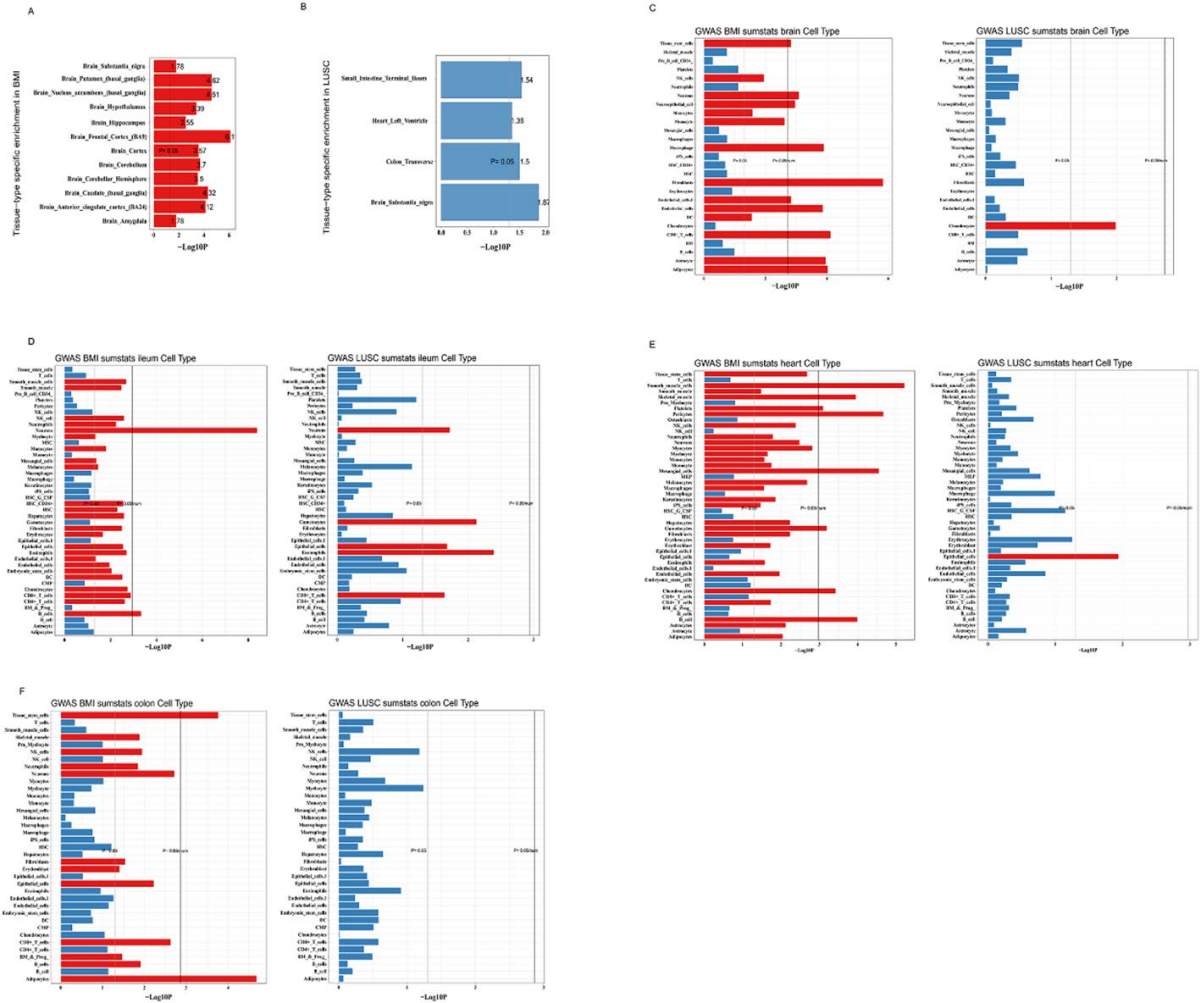
Tissue- and cell type–specific enrichment of SNP heritability for BMI and LUSC. **A** Tissue-level SNP heritability enrichment for BMI across 53 GTEx tissues estimated using LDSC-SEG. The x-axis shows tissues grouped by organ system, and the y-axis shows the estimated enrichment coefficient (log-scaled or standardized). Tissues surpassing the significance threshold (FDR-adjusted *P* < 0.05) are highlighted. **B** Tissue-level SNP heritability enrichment for LUSC using the same LDSC-SEG framework. The x-axis indicates GTEx tissues and the y-axis indicates enrichment coefficients, as in panel (**A**). Tissues with significant enrichment (FDR-adjusted *P* < 0.05) are labelled, illustrating the overlap and differences in tissue-specific genetic architecture between BMI and LUSC. **C**–**G** Cell type–specific SNP heritability enrichment for BMI and LUSC across single-cell RNA sequencing datasets from brain, ileum, heart, and colon, estimated using MAGMA-based cell typing. For each tissue, the left panel shows enrichment for BMI and the right panel shows enrichment for LUSC. The y-axis lists cell types (for example, neurons, epithelial cells, eosinophils, CD8⁺ T cells), and the x-axis shows –log₁₀(P) values for the association between cell type–specific expression and SNP heritability. Cell types with *P* < 0.05 are indicated in red, highlighting those most strongly enriched for the genetic signal of BMI and/or LUSC. *BMI* body mass index, *LUSC* lung squamous cell carcinoma, *SNP* single nucleotide polymorphism, *LDSC-SEG* linkage disequilibrium score regression applied to specifically expressed genes, *MAGMA* Multi-marker Analysis of GenoMic Annotation, *scRNA-seq* single-cell RNA sequencing, *GTEx* Genotype-Tissue Expression

We next investigated tissue-level SNP heritability enrichment using LDSC-SEG to identify tissues in which genes with increased expression are enriched for BMI- and LUSC-associated SNPs. As shown in Fig. 4A, BMI exhibited significant SNP heritability enrichment in multiple tissues, with the strongest signals observed in several brain regions, consistent with prior evidence implicating central nervous system pathways in obesity susceptibility (Table S8). For LUSC, LDSC-SEG revealed significant enrichment in a partially overlapping but distinct set of tissues, including brain, colon, heart, and small intestine (Fig. 4B; Table S8). The co-enrichment of BMI and LUSC in brain tissue suggests that some shared genetic influences may act through neural pathways, whereas the additional LUSC enrichment in gastrointestinal and cardiac tissues highlights the broader systemic impact of its genetic architecture.

### Cell-level SNP heritability enrichment showed co-enrichment of SNPs from BMI and LUSC in the epithelial cells and neurons

To further dissect the cell type–specific basis of the genetic signals identified at the tissue level, we analyzed scRNA-seq datasets from the brain, heart, ileum and colon using MAGMA-based cell typing. As summarized in Fig. 4C–G, we observed significant enrichment of BMI- and LUSC-associated SNPs in specific cell types within these tissues.

In the ileum dataset in particular, both BMI and LUSC showed significant co-enrichment in neurons, epithelial cells, eosinophils and CD8 + T cells (*P* < 0.05), as indicated by the red bars in Fig. 4E. In contrast, no clear co-enrichment across BMI and LUSC was detected in heart, colon or brain cells (Fig. 4C, D, F and G). These findings may indicate that neurons and epithelial cells in the ileum, together with selected immune cell subsets, may play an important role in mediating the shared genetic effects on obesity and LUSC.

### *MFAP1* was identified as a shared functional gene for BMI and LUSC

After establishing a shared genetic architecture at the genome, tissue, and cell type levels, we next sought to identify specific genes that may underlie the BMI–LUSC relationship. Using SMR based on whole-blood eQTL data from eQTLGen, we identified 11 shared risk genes with PSMR < 0.005 and PHEIDI > 0.05 (Figure S2). Among these, seven genes mapped to genomic regions highlighted by the cross-trait analyses (Table S9; Figure S3), including EPHB1, PRSS16, ZSCAN16, ZKSCAN8, CYP21A2, PDIA3, and MFAP1.

Subsequently, we performed colocalization analyses to further assess whether the GWAS and eQTL signals for these genes are driven by shared causal variants. As shown in Table S10 and Figure S3, *MFAP1* exhibited high colocalization with BMI (PPH4 = 0.883) and moderate colocalization with LUSC (PPH4 = 0.561), whereas *PDIA3*, *CYP21A2*, *PRSS16*, *ZSCAN16* and *EPHB1* did not show consistent evidence of colocalization with both traits (PPH4 < 0.5, Table S10). Based on the convergence of SMR and colocalization results, *MFAP1* was prioritized as a key shared functional gene linking BMI and LUSC.

### Phenome-wide MR revealed that increasing MFAP1 is associated with connective tissue–and other disease phenotypes

Finally, we conducted a phenome-wide MR using data from the eQTLGen phase II and the Iceland 4907 pQTL dataset as exposures to investigate possible side effects of therapeutically targeting *MFAP1* at the mRNA and protein levels, respectively. In summary, genetically higher *MFAP1* expression showed limited but notable associations with several disease phenotypes, including dermatologic conditions and sleep apnea at the mRNA level, and arthropathies at the protein level (Figure S4). These findings suggest that modulation of *MFAP1* may influence connective tissue–related and other clinical phenotypes, and such potential effects would need to be carefully evaluated in future therapeutic development.

## Discussion

In this study, we revealed the potential causal relationship between BMI and LUSC at the genetic level and identified their shared risk regions. We found that most of the shared SNPs were enriched in epithelial cells and neurons. MFAP1 was identified as an important shared genetic component of the BMI-LUSC relationship, and potential side-effects of increasing MFAP1 were preliminarily investigated.

We found strong genetic correlations between BMI and LUSC using LDSC, GNOVA and ρ-HESS. Cross-trait meta-analyses identified 350 SNPs in MTAG and CPASSOC analyses, of which 97 SNPs were located in significant genomic regions estimated by ρ-HESS. We used two different statistical analysis methods (MTAG and CPASSOC) to minimize bias due to possible sample overlap. In addition to the IVW method, we applied multiple MR approaches, including MR-EGGER, weighted median, weighted mode and GSMR, to explore the causal relationship between BMI and LUSC [39–42]. The consistency of results across methods supports the robustness of the findings, and sensitivity analyses further supported their validity. In particular, we excluded smoking-related SNPs when selecting SNPs to reduce confounding by smoking. Overall, we found that high BMI can increase the risk of LUSC, whereas LUSC has minimal impact on BMI.

We also applied LDSC-SEG to analyze the GTEx data for tissue-level SNP heritability enrichment [31]. We identified 12 tissues with significant SNP heritability enrichment for BMI, mainly in brain regions. Growing evidence suggests that susceptibility to obesity is distributed across multiple brain regions and is strongly associated with structural abnormalities, which corroborates our results [11]. For LUSC, enrichment was mainly observed in four tissues: brain, colon, heart, and small intestine, indicating a broad systemic influence.

Based on publicly available single-cell data from GEO, MAGMA analysis showed that shared SNPs were specifically enriched in neurons and epithelial cells [32]. Previous researches have shown that obesity may drive neurological dysfunction and neurodegeneration, and that abnormal neural activities also play an important role in tumor progression [43, 44]. LUSC arises from transformation of squamous cells in the lining of the respiratory tract, and several recent studies have provided compelling evidence that changes in epithelial barrier function and inflammation are associated with, and may even lead to altered regulation of body weight and glucose homeostasis [45]. Thus, these two diseases may be intensely connected through genetic changes in epithelial and nervous tissue.

We inferred that *MFAP1* is a possible shared gene for obesity and LUSC using SMR, co-localization and complementary integrative analyses [33]. We found that the expression of this gene is elevated in squamous lung cancer and shows strong colocalization with both obesity- and LUSC-related genetic signals. *MFAP1* is a microvillus-associated gene [46], and according to the Human Protein Atlas, patients with LUSC who have higher *MFAP1* expression generally survive longer than those with lower expression. At present, however, the molecular mechanism linking *MFAP1* to the development of obesity and LUSC have not been elucidated by functional experiments, which we believe is an important direction for future research. Consistent with this, our phenome-wide MR analyses indicated limited but notable associations between genetically higher *MFAP1* levels and several disease phenotypes, including dermatologic disorders, sleep apnea, and arthropathies. These potential connective tissue– and respiratory-related effects highlight that any therapeutic strategy targeting *MFAP1* would need to balance putative benefits on LUSC risk against the possibility of adverse effects on other organ systems.

Beyond the statistical evidence, several biological features of *MFAP1* make it a plausible mediator of the BMI–LUSC relationship. *MFAP1* encodes a microfibril-associated protein that participates in extracellular matrix organization and microfibrillar assembly, processes that are closely linked to tissue elasticity, stromal remodeling, and epithelial–mesenchymal interactions [46, 47]. Dysregulation of extracellular matrix dynamics has been implicated both in adipose tissue expansion and chronic low-grade inflammation in obesity [48], and in tumor invasion, angiogenesis, and immune evasion in lung cancer [49]. It is therefore conceivable that genetically driven variation in *MFAP1* could influence obesity-related remodeling of the pulmonary and systemic microenvironment and, in turn, susceptibility to LUSC. Our findings extend previous multi-omics and phenomics studies that have used transcriptomic profiling and network analyses to identify hub genes and prognostic signatures in non-small cell lung cancer by showing that MFAP1 emerges as a shared gene at the level of germline genetic architecture linking obesity and LUSC [13, 15]. Nevertheless, these interpretations remain hypothesis-generating and require experimental confirmation. Future studies should include functional perturbation of *MFAP1* in lung epithelial cells and adipocyte models, in-depth characterization of downstream signaling and extracellular matrix changes, as well as in vivo validation in appropriate mouse models, to clarify the causal pathways and evaluate whether *MFAP1* represents a viable and safe therapeutic target.

The number of obese people worldwide is still rising non-stop [50], and lung cancer is one of the most commonly diagnosed cancers and the leading cause of cancer-related deaths worldwide, with an estimated 2 million new cases and 10–76 million deaths per year [51]. Our study elucidated the common genetic structure between BMI and squamous lung cancer and identified shared functional genes, which will provide very valuable directions for the future treatment and prevention of the two co-morbidities or even individually.

This study has several limitations that should be acknowledged. First, all GWAS datasets included in our analyses were derived exclusively from individuals of European ancestry. As a result, the generalizability of our findings to other ancestral populations remains limited, and the observed genetic relationships between BMI and LUSC may differ across ethnic groups due to population-specific allele frequencies and environmental exposures. Future studies incorporating multi-ethnic cohorts will be essential to validate the cross-population robustness of our conclusions. Second, although we performed multiple complementary analyses and sensitivity tests to strengthen the reliability of our results, we were unable to conduct independent replication in external datasets because publicly available LUSC GWAS summary statistics from non-European populations are scarce. The absence of an external validation cohort reduces the ability to confirm the reproducibility of the identified shared loci and the inferred causal relationship. Third, the identification of *MFAP1* as a shared genetic factor between BMI and LUSC is based on integrative statistical evidence from SMR and colocalization analyses. However, these findings remain exploratory, and no functional or experimental studies have yet confirmed the mechanistic roles of *MFAP1* in adiposity-related biological pathways or squamous lung carcinogenesis. The biological plausibility inferred here therefore requires further validation using in vitro and in vivo models. Finally, although we attempted to reduce potential confounding and pleiotropy by applying multiple MR methods and pleiotropy-robust sensitivity analyses, residual horizontal pleiotropy cannot be entirely excluded. Additionally, potential sample overlap between BMI and LUSC GWAS datasets may introduce bias despite our use of constrained intercepts and complementary methods to mitigate this risk. Future studies with pedigree-based datasets or individual-level data may help further clarify these issues.

## Supplementary Information

Below is the link to the electronic supplementary material.

Supplementary Material 1.

Supplementary Material 2.

Supplementary Material 3.

## Data Availability

All data used in this study are publicly available. Datasets for the BMI and LUSC were obtained from IEU OpenGWAS project ( [https://gwas.mrcieu.ac.uk/](https:/gwas.mrcieu.ac.uk) ), their GWAS ID were ieu-b-5118 and ieu-a-989, separately. The Single-cell RNA sequencing gene expression data were available from https://www.ncbi.nlm.nih.gov/geo/ and [https://singlecell.broadinstitute.org/single_cell](https:/www.singlecell.broadinstitute.org/single_cell) . The accession numbers of scRNA-seq were: brain (GSE162631), colon (GSE166555), small intestine terminal ileum (GSE242086), heart (SCP498, Single Cell Portal).The phenome-wide GWAS summary data were obtained from SAIGE GWAS at [https://www.leelabsg.org/resources](https:/www.leelabsg.org/resources) . Whole-blood cis-eQTL summary statistics was available from eQTLGen (https://eqtlgen.org/).All software and packages used are publicly available. The codes used in this study can be found at: LDSC: [https://github.com/bulik/ldsc](https:/github.com/bulik/ldsc) .PLINK: https://www.cog-genomics.org/plink/1.9.MTAG: [https://github.com/JonJala/mtag](https:/github.com/JonJala/mtag) .CPASSOC: http://hal.case.edu/∼xxz10/zhuweb/.GSMR: [http://cnsgenomics.com/software/gsmr/](http:/cnsgenomics.com/software/gsmr) .TwoSampleMR: [https://mrcieu.github.io/TwoSampleMR/](https:/mrcieu.github.io/TwoSampleMR) .SMR: [https://cnsgenomics.com/software/smr/#Overview](https:/cnsgenomics.com/software/smr) .LDSC-SEG: [https://github.com/bulik/ldsc/wiki/Cell-type-specificanalyses](https:/github.com/bulik/ldsc/wiki/Cell-type-specificanalyses) .MAGMA Celltyping: https://neurogenomics.github.io/MAGMA_ Celltyping.
