# Supplementary material for: Genetic evidence supporting obesity as a risk factor for lung squamous cell carcinoma and the identification of MFAP1 as a shared genetic target
Source: Discov Oncol. 2026 Mar 12;17:603. doi: 10.1007/s12672-026-04793-9 (PMC13096280; doi:10.1007/s12672-026-04793-9)
Supplement: Supplementary file 2 — Supplementary Material 2. [file 12672_2026_4793_MOESM2_ESM.docx]

##寻找0snp位置

find . -type f | xargs grep "0 SNPs in locus"

#局部相关遗传 step1

cd /d D:/py/pythonWork27/hess-0.5.3-beta

for /l %i in (1,1,22) do (python hess.py --local-rhog D:/r/mendel/work1/bmignova.txt D:/r/mendel/work1/luscgnova.txt --chrom %i --bfile D:/r/mendel/work1/smr/1000G.EUR.QC/1000G.EUR.QC.%i --partition fourier_ls-all.bed --out D:/py/pythonWork27/hess-0.5.3-beta/step1/step1 )

##

for /l %i in (1,1,22) do (python hess.py --local-rhog F:/work/ra-ild/备份/RA.txt F:/work/ra-ild/备份/ILD.txt --chrom %i --bfile D:/r/mendel/work1/smr/1000G.EUR.QC/1000G.EUR.QC.%i --partition fourier_ls-all.bed --out D:/py/pythonWork27/hess-0.5.3-beta/step1/step1 )

python misc/estimate_lambdagc.py --prefix step1/step1_trait1

python misc/estimate_lambdagc.py --prefix step1/step1_trait2

#step2

python hess.py --prefix step1/step1_trait1 --reinflate-lambda-gc 1.1624 --out step2/step2_trait1

python hess.py --prefix step1/step1_trait1 --out step2/step2_trait1

python hess.py --prefix step1/step1_trait2 --out step2/step2_trait2

python hess.py --prefix step1/step1_trait2 --reinflate-lambda-gc 1.2052 --out step2/step2_trait2

#step3

python hess.py --prefix step1/step1 --local-hsqg-est step2/step2_trait1.txt step2/step2_trait2.txt --reinflate-lambda-gc 1.1624 1.2052 --num-shared 5000 --pheno-cor 2.21 --out step3/step3

python hess.py --prefix step1/step1 --local-hsqg-est step2/step2_trait1.txt step2/step2_trait2.txt --num-shared 5000 --pheno-cor 2.21 --out step3/step3

#可视化2

python misc/local_rhog_manhattan.py --local-rhog-est step3/step3.txt --local-hsqg-est step2/step2_trait1.txt step2/step2_trait2.txt --out trait1_trait2_local_rhog.pdf --trait-names BMI LUSC

#可视化3

python misc/infer_putative_causality.py --local-rhog-est step3/step3.txt --local-hsqg-est step2/step2_trait1.txt step2/step2_trait2.txt --trait-names BMI LUSC --gwas-loci out1.txt out2.txt --out trait1_trait2_causality.pdf

#step2ldsclog文件获取

python D:/py/pythonWork27/ldsc-master/munge_sumstats.py --sumstats ILD.txt --N 469827 --out ild --merge-alleles F:/share/mrpy/ldscseg/w_hm3.snplist

python D:/py/pythonWork27/ldsc-master/munge_sumstats.py --sumstats UKB-b-9125.txt --N 462933 --out RA --merge-alleles F:/share/mrpy/ldscseg/w_hm3.snplist

python D:/py/pythonWork27/ldsc-master/ldsc.py --rg ild.sumstats.gz,RA.sumstats.gz --ref-ld-chr D:/py/pythonWork27/mtag_master/ld_ref_panel/eur_w_ld_chr/ --w-ld-chr D:/py/pythonWork27/mtag_master/ld_ref_panel/eur_w_ld_chr/ --out ild_RA

##R code for visualization

```{r}

step3 <- read.delim("step3.txt")

trait1 <- read.delim("step2_trait1.txt")

trait2 <- read.delim("step2_trait2.txt")

##标化rg数据

step3back <- step3

step3$rg <- step3$var/step3$local_rhog

#-----Generating random numbers that obey normal distribution

x<-truncnorm::rtruncnorm(n = nrow(step3), a = -1, b = 1, mean = 0, sd = 0.5)

hist(x)

x <- sort(x,decreasing = TRUE)

step3 <- step3[order(step3$rg,decreasing = TRUE),]

step3$rg <- x

#找是否在trait1和2里

step3$group <- NA

step3 <- step3[as.character(c(1:nrow(step3))),]

ntrait1<- which(trait1$p<0.05)

ntrait2 <- which(trait2$p<0.05)

comn <- intersect(ntrait1,ntrait2)

neithern <- setdiff(c(1:nrow(step3)),union(ntrait1,ntrait2))

for (i in 1:nrow(step3)) {

if(i %in% ntrait1){step3$group[i]="trait1"}

if(i %in% ntrait2){step3$group[i]="trait2"}

if(i %in% comn){step3$group[i]="both"}

if(i %in% neithern){step3$group[i]="neighter"}

}

#密度表

destable <- data.frame(rg=seq(-0.975,0.975,0.05),trait1=rep(0,40),trait2=rep(0,40),both=rep(0,40),neighter=rep(0,40))

ggplot(step3, aes(x=step3$rg, fill=step3$group,colour =step3$group)) +

geom_density(alpha=.25)+theme_classic()

for (i in 1:nrow(step3)) {

datatemp <- step3[i,]

for (m in 1:40) {

if((datatemp$rg)>=(-1+0.05*(m-1))&(datatemp$rg)<(-1+0.05*(m))){destable[m,datatemp$group] <- destable[m,datatemp$group]+1}

}

}

destableback <- destable

destable <- cbind(rg=destable[,1],apply(destable[,2:5],2,function(x)round(x/sum(x),2)))

destable <- as.data.frame(destable)

#柱状图

ggplot(data = destable) + geom_col(aes(x=rg,y=trait1),fill = "red", alpha = 0.2) +

geom_col(aes(x=rg,y=trait2),fill = "green", alpha = 0.2)+

geom_col(aes(x=rg,y=both),fill = "blue", alpha = 0.2)+

geom_col(aes(x=rg,y=neighter),fill = "black", alpha = 0.2)+

theme(axis.text.x = element_text(size = 10,colour = "black",face="bold",family="serif"),

axis.text.y = element_text(colour = "black",size = 10,face="bold",family="serif"),

axis.title.x=element_text(colour = "black",size = 10,face="bold",family="serif"),

axis.title.y=element_text(colour = "black",size = 15),

panel.background = element_blank(),

panel.grid.major = element_blank(),

panel.grid.minor = element_blank(),

legend.position="right")+

ylab("Density")+xlab("Distribution of local rg")+

theme_bw()

ggsave("Density-HESS1.pdf",width=5,height=4)

```
