# Supplementary material for: Genetic evidence supporting obesity as a risk factor for lung squamous cell carcinoma and the identification of MFAP1 as a shared genetic target
Source: Discov Oncol. 2026 Mar 12;17:603. doi: 10.1007/s12672-026-04793-9 (PMC13096280; doi:10.1007/s12672-026-04793-9)
Supplement: Supplementary file 3 — Supplementary Material 3. [file 12672_2026_4793_MOESM3_ESM.docx]

**Supplementary figures**

**
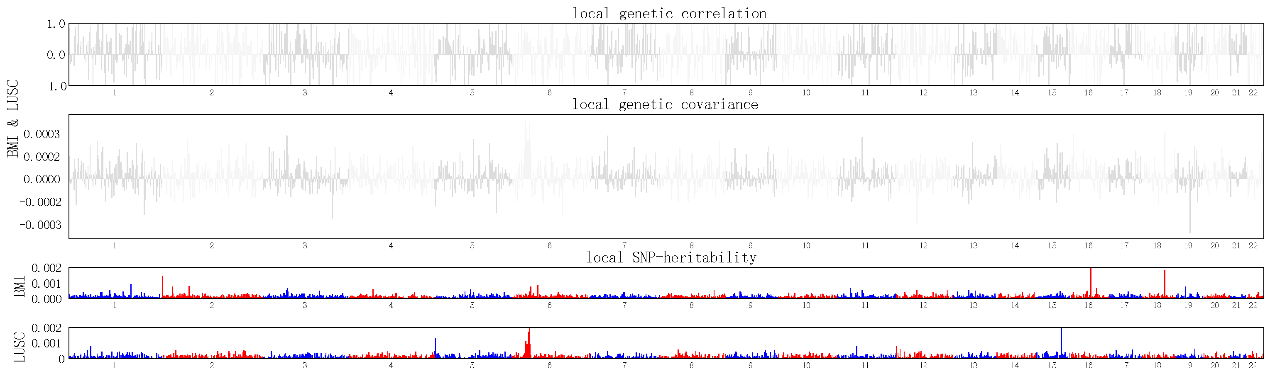
**

Figure S1: Local genetic correlations between BMI and LUSC revealed by ρ-HESS (Heritability Estimation from Summary Statistics). Blue: significant local genetic correlation for odd chromosomes. Red: significant local genetic correlation for even chromosomes. BMI, body mass index; LUSC, lung squamous cell carcinoma.


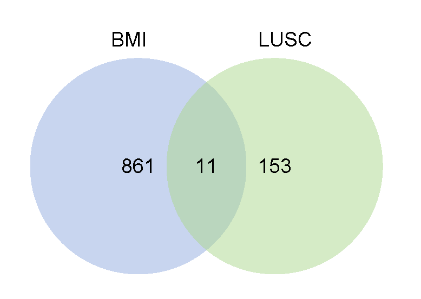


Figure S2: The ween plot of significant genes in the SMR analysis of eQTLs on BMI and LUSC. The intersection part are their common genes. Aberrations: BMI, body mass index; LUSC, squamous cell lung cancer.


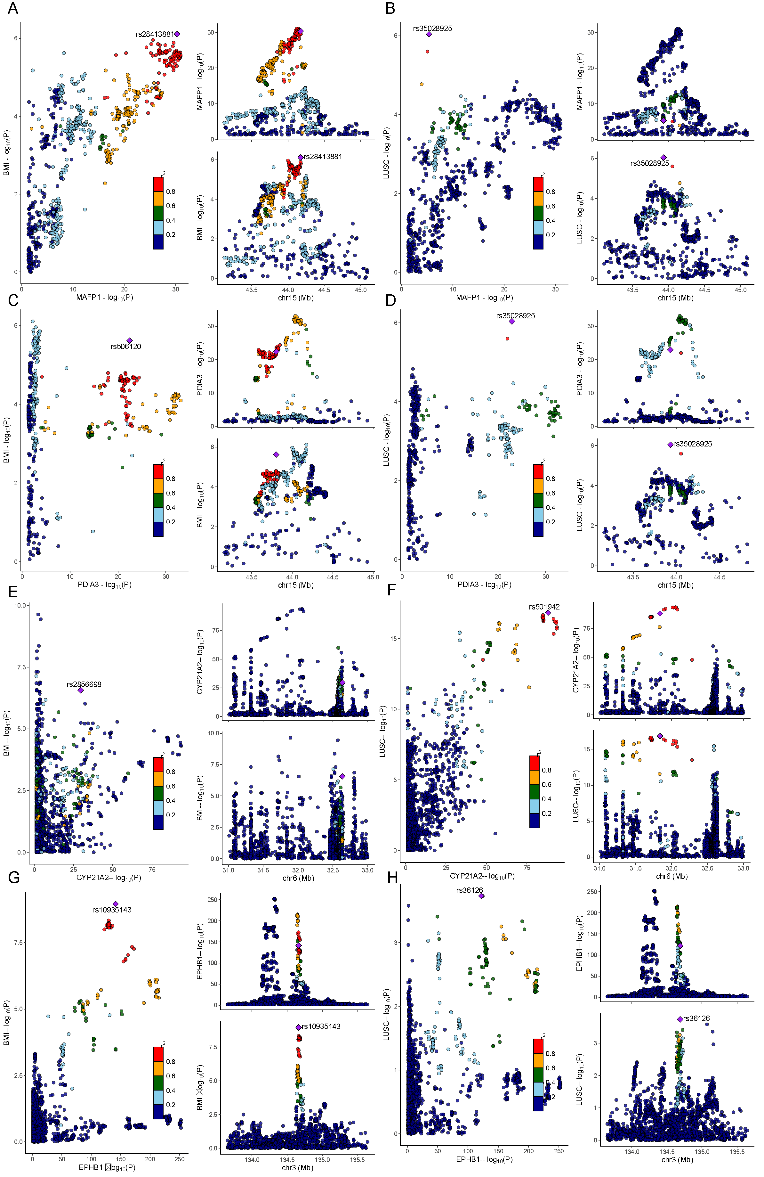


Figure S3：Colocalization analysis of potential target genes and corresponding traits. In each subgraph, the figure on the left side: Negative log10 P-values of traits are displayed on the y axis, negative log10 P-values of genes are displayed on the x axis. The figure on the right side: Negative log10 P-values of genes are displayed on the y axis, position of the variant is displayed on the x axis. A dot represents a variant. Colors represent different section of r2.

A: Colocalization analysis between eQTL signal in blood for *MFAP1* and BMI.

B: Colocalization analysis between eQTL signal in blood for *MFAP1* and LUSC.

C: Colocalization analysis between eQTL signal in blood for *PDIA3* and BMI.

D: Colocalization analysis between eQTL signal in blood for *PDIA3* and LUSC.

E: Colocalization analysis between eQTL signal in blood for *CYP21A2* and BMI.

F: Colocalization analysis between eQTL signal in blood for *CYP21A2* and LUSC.

G: Colocalization analysis between eQTL signal in blood for *EPHB1* and BMI.

H: Colocalization analysis between eQTL signal in blood for *EPHB1* and LUSC.

I: Colocalization analysis between eQTL signal in blood for *ZKSCAN8* and BMI.

J: Colocalization analysis between eQTL signal in blood for *ZKSCAN8* and LUSC.

K: Colocalization analysis between eQTL signal in blood for *ZSCAN16* and BMI.

L: Colocalization analysis between eQTL signal in blood for *ZSCAN16* and LUSC.

M: Colocalization analysis between eQTL signal in blood for *PRSS16* and BMI.

N: Colocalization analysis between eQTL signal in blood for *PRSS16* and LUSC.


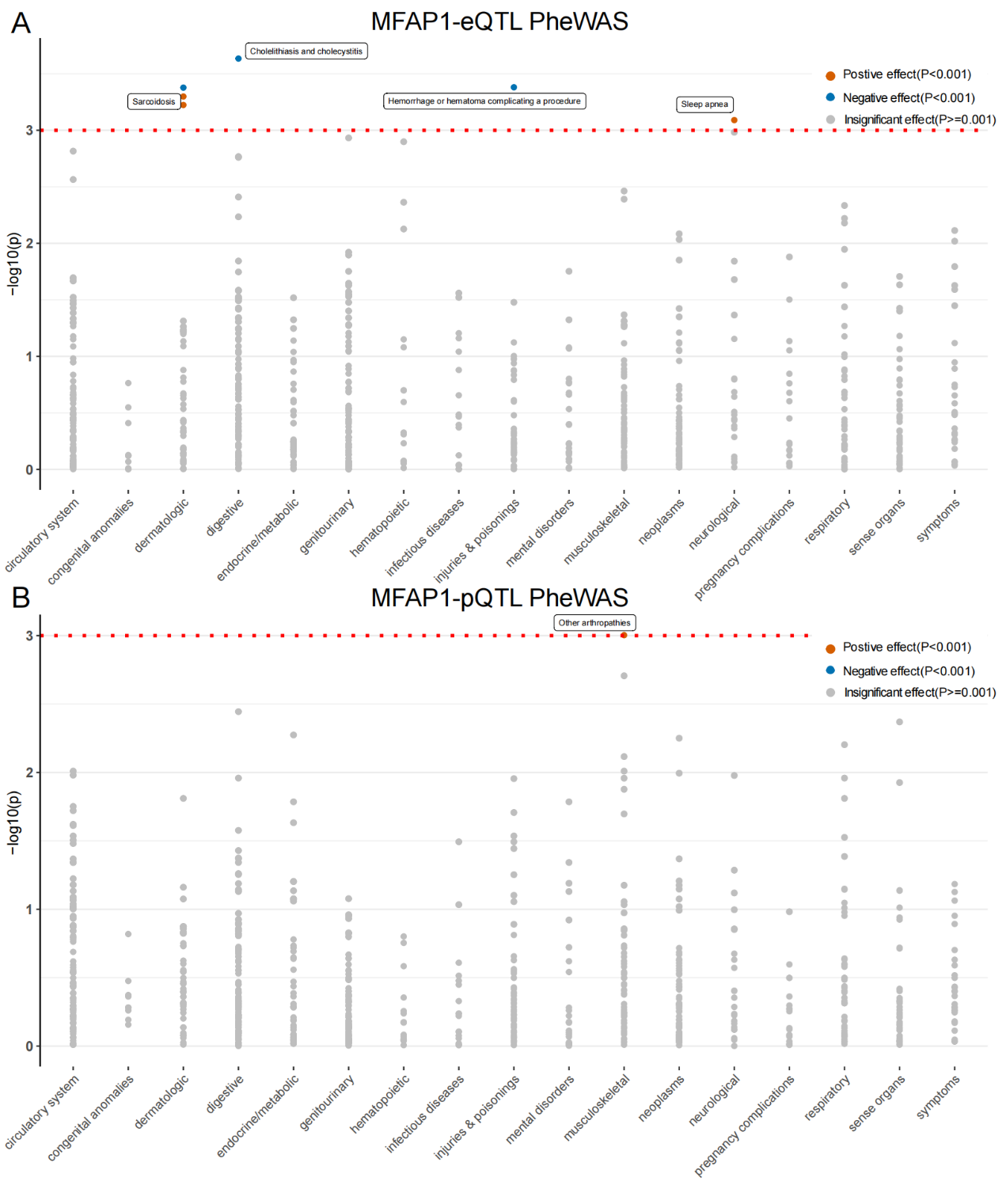


Figure S4: Manhattan plot for phenome-wide MR results of *MFAP1*. Negative log10 p-values are displayed on the y axis. A dot represents a disease trait, different color represents the effect direction and whether the effect was significant.
